# Protective Effects of *Ocimum gratissimum*Aqueous Extracts on HaCaT Cells Against UVC-Induced Inhibition of Cell Viability and Migration

**DOI:** 10.7150/ijms.54644

**Published:** 2021-03-12

**Authors:** Sheng-Huang Chang, Jer-Yuh Liu, Meen-Woon Hsiao, Hsin‐Ling Yang, Guan-Wei Wang, Je-Chiuan Ye

**Affiliations:** 1 Tsaotun Psychiatric Center, Ministry of Health and Welfare, Nantou, Taiwan.; 2 Graduate Institute of Biomedical Sciences, China Medical University, Taichung, Taiwan.; 3 Center for Molecular Medicine, China Medical University Hospital, Taichung, Taiwan.; 4 School of Applied Chemistry, Chung-Shan Medical University, Taichung, Taiwan; 5 Institute of Nutrition, College of Biopharmaceutical and Food Sciences, China Medical University, Taichung, Taiwan.; 6 Department of Bachelor's Degree Program for Indigenous Peoples in Senior Health and Care Management, National Taitung University, Taitung, Taiwan.; 7 Master Program in Biomedical Science, National Taitung University, Taitung, Taiwan.

**Keywords:** Ultraviolet C, Skin cells, proliferation, migration, *Ocimum gratissimum.*

## Abstract

Ultraviolet C (UVC) has been applied to treatment of infections in wounds for at least the last two decades, however, cells being treated can be damaged if exposure is prolonged, which calls for protective measures, such as drug or herbal pre-treatment, to minimize damage. *Ocimum gratissimum* contains plant polyphenols such as isoflavones and caffeic acid, which have antioxidant effects. We hypothesize that *Ocimum gratissimum* aqueous extracts (OGE) can inhibit UVC-induced oxidative damage on skin cells. In this study, HaCaT skin cells are used to test the protective effects of OGE on cell proliferation and migration after exposure to UVC radiation. Pretreatment with OGE (50~150μg/mL) before 40 J/m^2^ UVC exposure was able to restore survival from 32.25% to between 46.77% and 68.00%, and 80 J/m^2^ UVC exposure from 11.49% to between 19.07% and 43.04%. Morphological observation of primarily apoptotic cell death confirms the above findings. The flow cytometry analysis revealed that UVC increased the number of cells at the sub-G1 phase in a dose dependent manner, and when pre-treated with OGE the changes were partially reversed. Moreover, the wound healing test for observing migration showed that UVC 40-80 J/m^2^ decreased cell migration to 47-28% activity and 100 μg/mL OGE was able to restore cell activity to81-69% at day 3. Based on the above results, we suggest that OGE has a protective effect on UVC-induced inhibition of cell proliferation and migration of skin cells and thus has potential application in wound care.

## Introduction

Germicidal ultraviolet C (UVC) reduces the rate of surgical site infections in orthopedic surgical procedures 1. The exposure of all types of biological matter to electromagnetic radiation (light) in the UV spectrum (between 200 and 400 nm) can cause molecular changes 2, and prolonged exposure to UVC in the range of 200-280 nm can cause irreversible damage, and, in bacteria, UVC induces the dimerization of pyrimidine residues, which can interrupt DNA transcription, translation, and replication 3, thereby inactivating the bacteria 4, 5-7. This property of UVC has been applied to treat infections in wounds for at least the last two decades. However, the exposure of human cells to UVC can cause side effects in terms of the formation of mutagenic and cytotoxic DNA lesions, cell death, and even the initiation of skin cancer progression when exposure is further prolonged 8, 9. UVC exposure can produce a series of reactive molecules, such as superoxide radicals, hydrogen peroxide, and hydroxyl radicals, which reacts with membrane lipids, cellular proteins, and nucleic DNA, provokes physiological cellular responses, and leads to cell death 9, 10-12. Protective measures are required to minimize the damage done by this phenomenon to the cells being treated 13.

*Ocimum gratissimum* (OG) is a commonly used herbal ingredient in traditional Chinese medicine and widely distributed in tropical and warm temperate geolocations. OG aqueous extracts (OGE) have several therapeutic functions, including anti-inflammation 14, analgesic and spasmolytic 15, antidiarrheal 16, antiviral 17, and antihyperglycemic activities 17, 19. OGE also modulates the immune response 20 and possess anticancer 21-26 and antibacterial activities 27. OGE, with its many antioxidant components, can also protect body organs from free radical damage and oxidative stress 28-37. We speculated that OGE can prevent UVC damage, and that this property can be applied to wound care.

HaCaT cells or human spontaneously immortalized keratinocytes with full epidermal differentiation capacity is an in vitro keratinocyte model that is widely used as a skin cell damage model 38-40. In the present study, we aimed to examine the effects of OGE on UVC-induced inhibition of cell proliferation and migration in human HaCaT cells.

## Materials and methods

### Materials

3-(4,5-Dimethylthiazol-2-yl)-2,5-diphenyl-tetrazolium bromide (MTT) and penicillin andstreptomycin were purchased from Sigma (St. Louis, MO). Roswell Park Memorial Institute Medium (RPMI1640), fetal bovine serum (FBS), and trypsin-EDTA were purchased from Gibco BRL (Gaithersburg, MD). The human epidermal skin cell (HaCaT) were obtained from American Type Culture Collection (ATCC; Rockville, MD).

### Cell culture

The cells were cultured in DMEM-F12 and supplemented with 10% FBS and 100 μg/mL penicillin/streptomycin at 37 °C in a humidified atmosphere containing 5% CO_2_. The cells were seeded in culture plates and grown to approximately 80% confluence. Cells (4×10^4^cells/mL) were then transported to experiment culture plates and maintained at 37 °C in a humidified atmosphere containing 5% CO_2_.After 24 h, the cells were treated with OGE at the indicated concentrations with UVC at the indicated energy for the indicated hours andthen collected for the following analyses.

### OGE preparation

OGE preparations were performed in accordance with our standardized procedure 21, and polyphenolic content is checked before use. OG leaves were harvested and washed with distilled water, followed by homogenization with distilled water by using a Polytron homogenizer. The homogenate was boiled for 1 h and then filtered through two layers of gauze. The filtrate was centrifuged at 20000 g at 4 °C for15 min to remove insoluble pellets. Then the supernatant (OGE) was collected, lyophilized, and stored at -70 °C until use.

### UVC exposure

The UV 254 dose was measured by a sensor in an UV Cross-linker CL-1000 (UVP, Upland, CA). All HaCaT cells were washed with phosphate buffered saline (PBS), and exposed to UVC radiation for the indicated UV doses (i.e., 0, 40, 60 and 80 J/m^2^). The PBS was removed and recultured in fresh culture medium with or without OGE. All assays were performed for the indicated time periods.

### Cell morphology

The cells were cultured in a 6-well culture dish with medium, and different OGE concentrations (i.e., 0, 50, 100, 200, 400, and 500 μg/mL) were added the next day. On the next day, the cells were to irradiated with UVC light at 40, 60, and 80 J/m^2^ and observed with an inverted microscope 20 times after 24 h.

### Cell viability

Cell viability was determined by MTT assay after treatment of the cells with indicated OGE and UVC concentrations for an indicated duration. After the treatments, medium was removed, and cells were incubated with 0.5 mg/mL MTT(3-(4,5-dimethylthiazol-2-yl)-2,5-diphenyltetrazolium bromide) at 37 °C for 2 h. The viable cell number was directly proportional to the production of formazan, which was dissolved in isopropanol and determined by measuring the absorbance at 570nm by using a microplate reader (Spectra MAX 360 pc, Molecular Devices, Sunnyvale, CA).

### Flow cytometry (FSCS)

The cell cycle was analyzed by FSCS after treatment of the cells with indicated OGE concentration. After 24 h, the cells were exposed to indicated energy of UVC and then continued to be cultured for 24 h. All cells, that is, cells in the suspension and adherent cells, were collected, washed, and suspended in cold PBS. The cells were then fixed in chilled 75% methanol and stained with propidium iodide. Analysis was performed in the FACSCalibur flow cytometer running CellQuest (Becton Dickinson, San Jose, CA).

### Wound healing assay

HaCaT cells were grown to confluence on 6-well microplates. The cell was treated with the indicated OGE concentration. After 24 h, a linear scratch was made using a 2 mm-wide Cell Scratcher™, and the wells were washed once with phosphate buffered saline (PBS). Immediately after washing, the cells were exposed to indicated energy of UVC and then added the indicated OGE concentration again. The cells were then observed with inverted microscope 40 times at 0, 24, 48, or 72 h after the scratch.

### Statistical analysis

Data were expressed as mean ± SEM of the three independent experiments and analyzed using ANOVA. Student's *t*-test was used in two-group comparisons. p<0.05 was considered to be statistically significant.

## Results

### Toxicity of OGE in HaCaT cells

**Figure 1:**
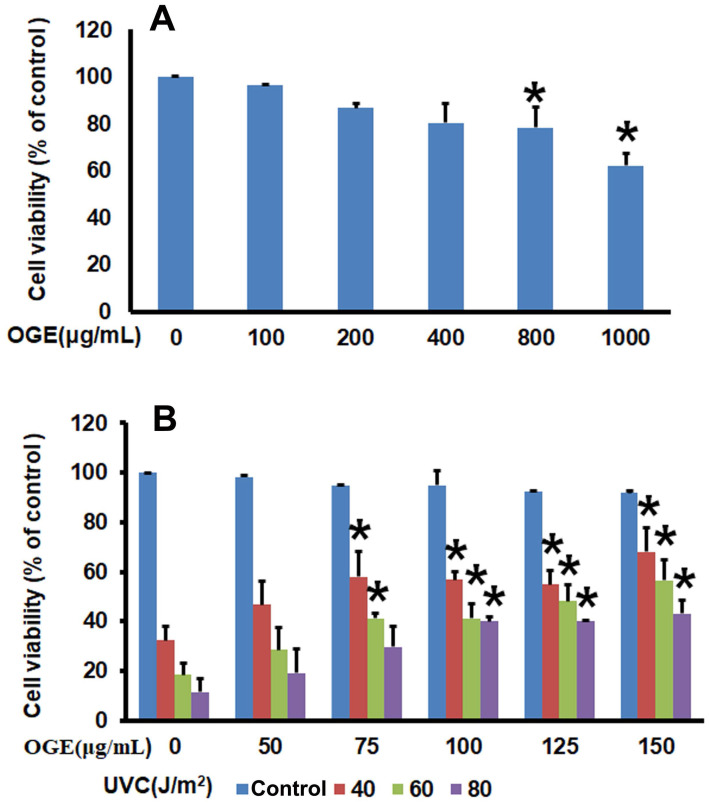
Effects of OGE on UVC-induced inhibition of cell viability of HaCaT cells. Seed cells in 6-well plate at 1.2×10^6^ cell/well. (A) After attachment, the cells were treated with indicated OGE concentration for 48 h, and the absorbance was measured after the cells were incubated with MTT for 2h. *p < 0.05, compared with the control group. (B) After attachment, the cells were treated with indicated OGE concentration for 24 h and then exposed to different doses of UVC. After 48 h, the absorbance was measured after the cells were incubated with MTT for 2h. *p < 0.05, compared with the control group. Data were expressed as mean ± SEM for 3 independent experiments.

After 0, 100, 200, 400, 800, and 1000 μg/mL OGE were added to the HaCaT human epidermal skin cells for 72 h, the analysis of cell survival rate by using MTT assay showed that OGE began to affect cell survival at above 800μg/mL OGE (Figure 1A).

### Effects of OGE on UVC-induced inhibition of HaCaT cell growth

After the cells were treated with OGE and UVC light source, the results showed that cell survival rate decreased to 32.25%, 18.49%, and 11.49% as a response to UVC energy (40, 60, and 80 J/m^2^, respectively). When the cells were pretreated with 50, 75, 100, 125, and 150 μg/mL OGE, the cell survival rates were recovered to46.77%, 54.84%, 56.85%, 57.90%, and 68.00%, respectively, for 40 J/m^2^;28.50%, 41.25%, 41.21%, 48.21%, and 56.47%, respectively, for 60 J/m^2^; and 19.07%, 29.60%, 40.00%, 40.14%, and 43.04%, respectively, for 80 J/m^2^ (Figure 1B). As shown above, OGE affected cell recovery, which plateaued at approximately 100 μg/mL.

### Effects of OGE on morphologic changes in UVC-induced cells

**Figure 2:**
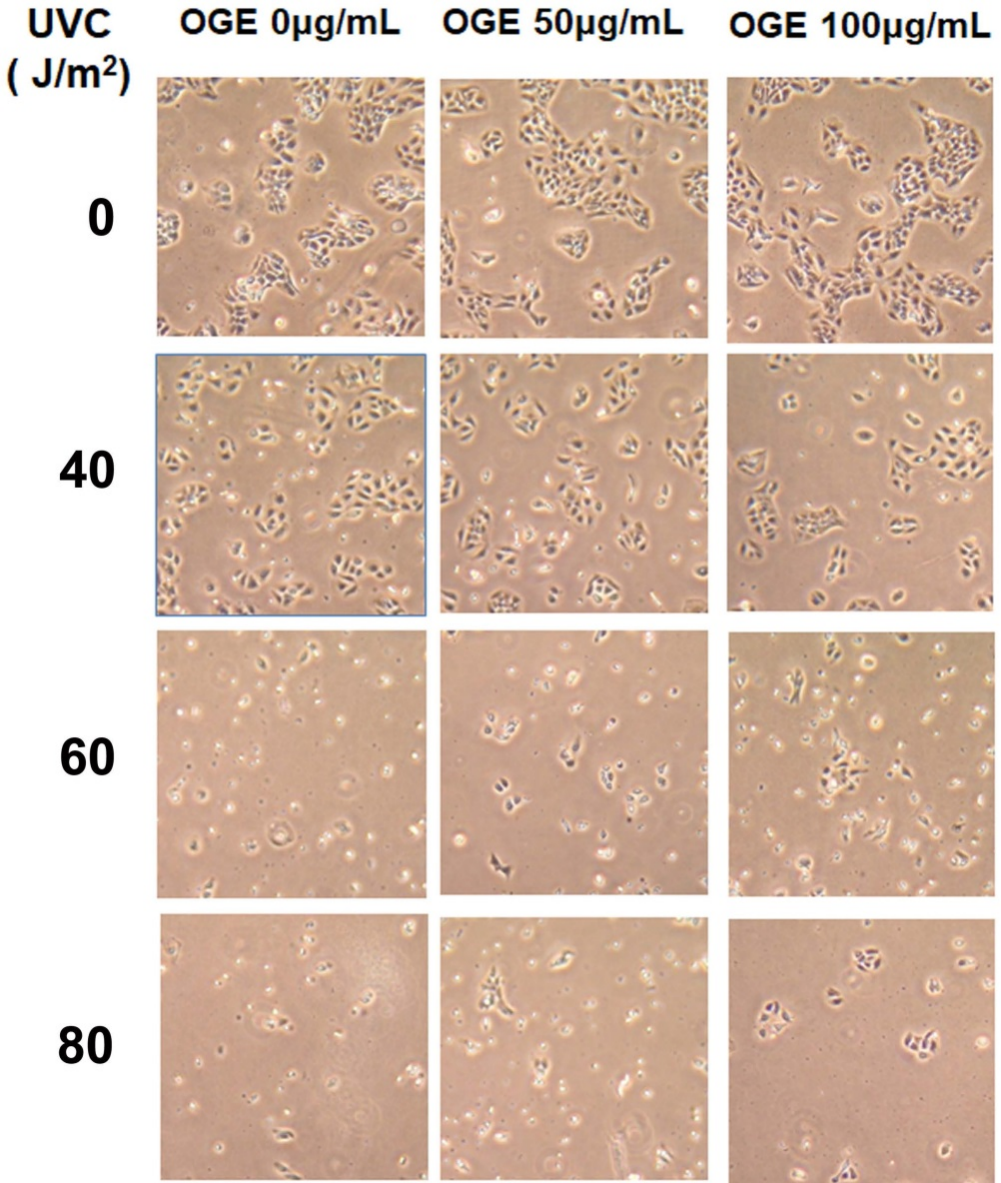
Effects of extract on UVC-induced changes in cell morphology of HaCaT cells pretreated with 0,50,100 μg/mL extract for 24 h and then exposed to 0,40,60, and 80 J/m^2^UVC. After another 24h, the cells were observed by a microscope with 40×magnification.

The cells were pretreated with 0, 50, and 100 μg/mL OGE for 24 h and then exposed to UVC light source at 0, 40, 60, and 80 J/m^2^. After the cells were cultured for 24 h, cell morphology was observed with a 20X microscope (Figure 2). Cell integrity began to show significant deterioration after 60 J/m^2^without OGE treatment, and OGE treatment protected the cells from morphologic change in a dose-dependent manner.

### Effects of OGE on UVC-induced changes in cell cycle of HaCaT cells

**Figure 3:**
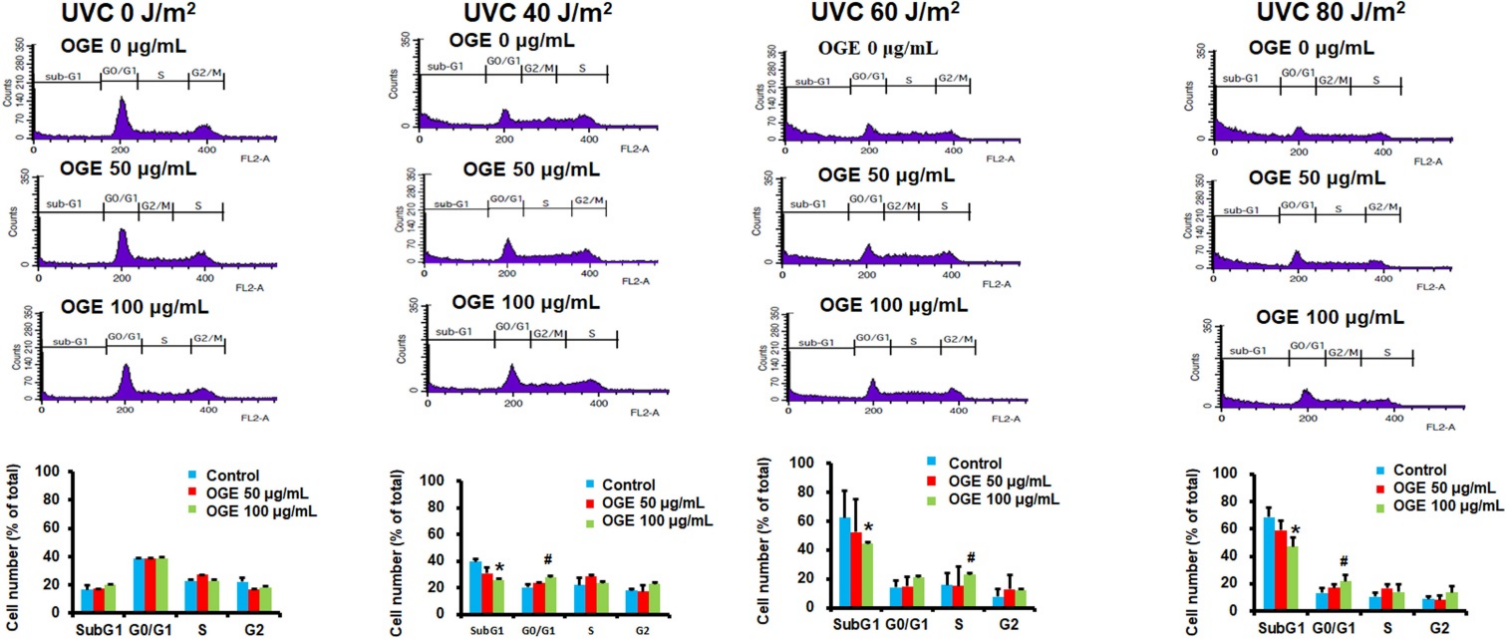
OGE protected against UVC-induced apoptosis in HaCaT cells. Cells were seeded in a 6-well plate at 1.2×10^6^ cells/well. After attachment, the cells were treated with indicated OGE concentration for 24 h and then exposed to different UVC doses. After 24 h, the cells were fixed with 99% methanol and stained propidium iodide and then analyzed by flow cytometry (FSCS). The percentage of cells within the hypodiploid DNA region was determined by FSCS. *p < 0.05, decrease as compared with the control group. ^#^p < 0.05, increase as compared with the control group. Data were expressed as mean ± SEM for 3 independent experiments.

The results above suggested that OGE can protect cells from UVC irradiation damage. To investigate whether the OGE protects cells against UVC-induced cell death, we further examined the changes in cell cycle. The results showed that 40, 60, and 80 J/m^2^UVC significantly increased the population in Sub-G1 phase to 221%, 332%, and 351%, respectively (Figure 3). The OGE pretreatment of 50 μg/mL decreased Sug-G1 population to 173%, 256%, and 294% and 100 μg/mL to 152%, 226%, and 233%. The same intensity of UVC significantly decreased the population in G1 phase to 56%, 27%, and 27%, respectively. The OGE pretreatment of 100 μg/mL to 70%, 51%, and 48%. The data here shows that G0/G1 does indeed increase, even if S or G2 did not. This result indicated that OGE had several protective effects against UVC-induced cell death.

### Effects of OGE on UVC-induced changes in wound healing process of HaCaT cells

**Figure 4:**
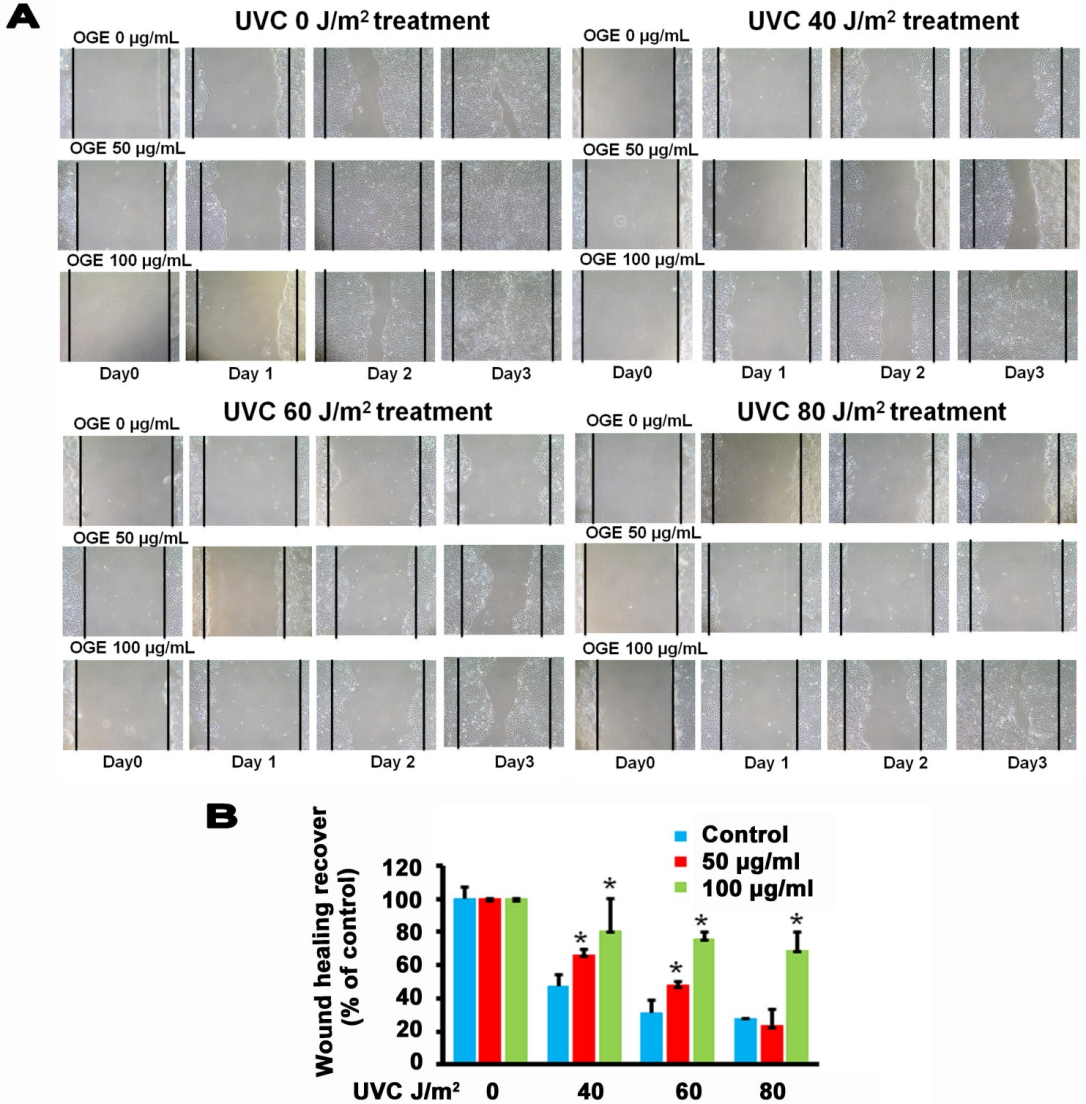
Effects of OGE on UVC-induced inhibition of HaCaT skin cell wound healing. After the cells were cultured until confluent, they were pretreated with 0, 50, and 100 μg/mL OGE for 24 h, then scratched with a pipette tip, and exposed to different UVC doses. (A) After the indicated times, the cells were photographed under a phase contrast microscope. (B) Statistical analysis of OGE on UVC-induced inhibition of HaCaT skin cell wound healing at day 3. *p < 0.05, compared with the control group. Data were expressed as mean ± SEM for 3 independent experiments.

The main components in the epidermis layer of the human skin are the keratinocytes, which are pathophysiologically related to wound healing but are also associated with other diseases such as psoriasis and atopic dermatitis 41, 42. Increasing the proliferation and migration of keratinocytes is beneficial to the complex process of wound healing, which involves unwounded keratinocytes near the wound site 43, 44. The data above supported the assertion that OGE exerts a protective effect against the UVC-induced inhibition of the proliferation of the human HaCaT cells. Then, we performed wound healing assay to test the effects of OGE on the UVC-induced inhibition of cell migration of human HaCaT cells. The results showed that 40, 60, and 80 J/m^2^ UVC decreased wound healing to 47%, 31%, and 28% activity, respectively (Figures 4). After the cells were pretreated with OGE, the cell activity was restored to 66%, 48%, and 23%for 50 μg/mL OGE and 81%, 76%, and 69% for 100 μg/mL OGE on day 3.

## Discussion

UV light that penetrates the skin reacts with the molecular oxygen (O_2_) in the cells of the mid-lower epidermis, and the donation of electron in the process generates a series of free radical compounds, from the superoxide anion (O_2_ꞏ^-^) to other resulting compounds, such as hydroxyl radicals 45, which provoke the cells with cellular responses and inhibit cell viability 9, 10-12. Research on protection against free radical damage has often introduced plant-based antioxidants that can scavenge free radicals, inhibit harmful cellular responses (e.g., inflammation), and stimulate other beneficial cellular responses, which increase cell survival. OGE is one such antioxidant sources that have been widely studied due to its function against oxidative stress 28-37. In this study, FSCS and MTT assay both showed that OGE restored subG1 and G0/G1phase cell count, even if S and G2 phase were not affected. This demonstrates survival, but proliferation is obviously hindered under the various levels of UVC exposure, thereby suggesting that OGE can protect against the UVC-induced inhibition of the HaCaT cell viability mediated through its antioxidant activities.

The cells are inherently equipped with antioxidant enzymes, including super oxide dismutase, catalase, and peroxidase 45, but continuous exposure to oxidative stress can deplete the enzyme supply. Thus, replenishment is needed to sustain the protective effect. For example, Oh et al. 46 reported that HaCaT keratinocytes can be protected against UV radiation-induced oxidative stress through the addition of ginsenoside Rb3 to enhance the total antioxidant enzyme levels and against UVC-triggered necrosis and inflammatory chemokine expression through plant polyphenol-enhanced antioxidant enzyme production 47. OGE is also known to enhance antioxidant enzyme production, and has been shown to inhibit CCl(4)-induced liver injuries in rats 48, 49. However, we suggest that OGE, in addition to antioxidant enzyme replenishment, may also increase the production of other supplemental components (i.e. cell-function enzymes) within the cells to decrease UVC-induced free radical damage, and we intend to further investigate this capacity.

The process of epithelialization describes the proliferation and migration of keratinocytes decreasing in the intermediate phase of inflammation 50 and is a crucial component of the wound healing processes, which include wound repair, sealing of the epidermal defect, and re-establishment of the barrier function 51-53.In this study, OGE demonstrated the above regardless of UV level. Similar to the activation of the PI3K/AKT pathway by OGE in H9c2 cells 30, which is an important signaling pathway related to migration 54, we suspect that OGE may activate the PI3K/AKT pathway in HaCaT cells which contributes to the reduction of the UVC-induced inhibition of migration.

Moreover, OGE is known to possess antibacterial effects 55. It has been proposed that its action against the bacteria may be due to the inhibition of cell wall formation in the bacteria resulting in a leakage of cytoplasmic constituents. We suggest this property makes OGE additionally suitable for application on UVC-based bacterial infection treatment.

## References

[1] Ritter MA, Olberding EM, Malinzak RA (2007). Ultraviolet lighting during orthopaedic surgery and the rate of infection. J Bone Joint Surg Am.

[2] Pfeifer GP, You YH, Besaratinia A (2005). Mutations induced by ultraviolet light. Mutat Res.

[3] Gurzadyan GG, Görner H, Schulte-Frohlinde D (1995). Ultraviolet (193, 216 and 254 nm) photoinactivation of Escherichia coli strains with different repair deficiencies. Radiat Res.

[4] Narita K, Asano K, Morimoto Y (2018). et al. Disinfection and healing effects of 222-nm UVC light on methicillin-resistant Staphylococcus aureus infection in mouse wounds. J Photochem Photobiol B.

[5] Dai T, Vrahas MS, Murray CK (2012). et al. Ultraviolet C irradiation: an alternative antimicrobial approach to localized infections?. Expert Rev Anti Infect Ther.

[6] Chang JC, Ossoff SF, Lobe DC (1985). et al. UV inactivation of pathogenic and indicator microorganisms. Appl Environ Microbiol.

[7] Tianhong D, Barbara Garci, Clinton K (2012). et al. UVC Light Prophylaxis for Cutaneous Wound Infections in Mice. Antimicrobial Agents and Chemotherapy.

[8] Pfeifer GP, Besaratinia A (2012). UV wavelength-dependent DNA damage and human non-melanoma and melanoma skin cancer. Photochem Photobiol Sci.

[9] Girotti A.W (1998). Lipid hydroperoxide generation, turnover, and effector action in biological systems. J. Lipid Res.

[10] Shih MF, Cherng YJ (2012). Protective effects of Chlorella-derived peptide against UVC-induced cytotoxicity through inhibition of caspase-3 activity and reduction of the expression of phosphorylated FADD and cleaved PARP-1 in skin fibroblasts. Molecules.

[11] Jiang W, Ananthaswamy HN, Muller HK (1999). et al. p53 protects against skin cancer induction by UV-B radiation. Oncogene.

[12] Wang SC, Chen SF, Lee YM (2013). et al. Baicalin scavenges reactive oxygen species and protects human keratinocytes against UVC-induced cytotoxicity. *in vivo*.

[13] Dai T, Vrahas MS, Murray CK (2012). Ultraviolet C irradiation: an alternative antimicrobial approach to localized infections?. Expert Rev Anti Infect Ther.

[14] Lin CC, Lin JK, Chang CH (1995). Evaluation of hepatoprotective effects of “Chhit-Chan-Than” from Taiwan. Pharm. Biol.

[15] Aziba PI, Bass D, Elegbe Y (1999). Pharmacological investigation of Ocimum gratissimum in rodents. Phytother. Res.

[16] Ilori M, Sheteolu AO, Omonigbehin EA (1996). et al. Antidiarrhoeal activities of Ocimum gratissimum (Lamiaceae). J. Diarrhoeal Dis. Res.

[17] Ayisi NK, Nyadedzor C (2003). Comparative in vitro effects of AZT and extracts of Ocimum gratissimum, Ficus polita, Clausena anisata, Alchornea cordifolia, and Elaeophorbia drupifera against HIV-1 and HIV-2 infections. Antiviral Res.

[18] Aguiyi JC, Obi CI, Gang SS (2000). et al. Hypoglycaemic activity of Ocimum gratissimum in rats. Fitoterapia.

[19] Casanova LM, da Silva D, Sola-Penna M (2014). et al. Identification of chicoric acid as a hypoglycemic agent from Ocimum gratissimum leaf extract in a biomonitoring in vivo study. Fitoterapia.

[20] Atal CK, Sharma ML, Kaul A (1986). et al. Immunomodulating agents of plant origin. I: Preliminary screening. J. Ethnopharmacol.

[21] Chen HM, Lee MJ, Kuo CY (2011). et al. Ocimum gratissimumaqueous extract induces apoptotic signalling in lung adenocarcinoma cell A549. Evid Based Complement Alternat Med.

[22] Lin CC, Chao PY, Shen CY (2014). Novel Target Genes Responsive to Apoptotic Activity by Ocimum gratissimum in Human Osteosarcoma Cells. Am J Chin Med.

[23] Xia M, Yu H, Gu S (2014). p62/SQSTM1 is involved in cisplatin resistance in human ovarian cancer cells via the Keap1-Nrf2-ARE system. Int J Oncol.

[24] Halatsch ME, Löw S, Mursch K (2009). Candidate genes for sensitivity and resistance of human glioblastoma multiforme cell lines to erlotinib. J Neurosurg.

[25] Nangia-Makker P, Raz T, Tait L (2013). Ocimum gratissimum retards breast cancer growth and progression and is a natural inhibitor of matrix metalloproteases. Cancer Biol Ther.

[26] Ekunwe SI, Hall SM, Luo X (2013). Fractionated Ocimum gratissimum leaf extract inhibit prostate cancer (PC3·AR) cells growth by reducing androgen receptor and survivin levels. J Health Care Poor Underserved.

[27] Evwierhurhoma E, Ugwu MC, Eze CO (2015). Antibacterial Evaluation of Aqueous and Ethanol Extracts of Ocimum gratissimum and Carica papaya. Annual Research & Review in Biology.

[28] George S, Chaturvedi P (2009). A comparative study of the antioxidant properties of two different species of Ocimum of southern Africa on alcohol-induced oxidative stress. J. Med. Food.

[29] Li PC, Chiu YW, Lin YM (2012). et al. Herbal Supplement Ameliorates Cardiac Hypertrophy in Rats with CCl4-Induced Liver Cirrhosis. Evid Based Complement Alternat Med.

[30] Lee MJ, Chen HM, Tzang BS (2011). et al. Ocimum gratissimum aqueous extract protects H9c2 myocardiac cells from H2O2-induced cell apoptosis through Akt signalling. Evid Based Complement Alternat Med.

[31] Chiu CC, Huang CY, Chen TY (2012). Beneficial Effects of Ocimum gratissimum Aqueous Extract on Rats with CCl4-Induced Acute Liver Injury. Evid Based Complement Alternat Med.

[32] Chang HC, Chiu YW, Lin YM (2014). Herbal supplement attenuation of cardiac fibrosis in rats with CCl4-induced liver cirrhosis. Chin. J. Physiol.

[33] Chiu YW, Chao PY, Tsai CC (2014). Ocimum gratissimum is effective in prevention against liver fibrosis in vivo and in vitro. Am J Chin Med.

[34] Chen YH, Chiu YW, Shyu JC (2015). et al. Protectiveeffects of Ocimum gratissimumpolyphenol extract on carbon tetrachloride-induced liver fibrosis in rats. Chin J Physiol.

[35] Chao PY, Lin JA, Ting WJ (2016). et al. Ocimum gratissmum aqueous extract reduces plasma lipid in hypercholesterol-fed hamsters. Int J Med Sci.

[36] Chao PY, Lin JA, Ye JC (2017). et al. Attenuation of oxidative stress-Induced cell apoptosis in schwann RSC96 cells by Ocimum gratissimumaqueous extract. Int J Med Sci.

[37] Chao PY, Chiang TI, Chang IC (2017). et al. Amelioration of estrogen-deficiency-induced obesity by Ocimum gratissimum. Int J Med Sci.

[38] Chao PY, Chiang TI, Chang IC (2017). Amelioration of estrogen-deficiency-induced obesity by Ocimum gratissimum. Int J Med Sci.

[39] López-García J, Lehocký M, Humpolíček P (2014). et al. HaCaT Keratinocytes Response on Antimicrobial Atelocollagen Substrates: Extent of Cytotoxicity, Cell Viability and Proliferation. J Funct Biomater.

[40] Boukamp P, Petrussevska RT, Breitkreutz D (1988). et al. Normal keratinization in a spontaneously immortalized aneuploid human keratinocyte cell line. J Cell Biol.

[41] Leng X, Shang J, Gao D (2018). et al. Low-intensity pulsed ultrasound promotes proliferation and migration of HaCaT keratinocytes through the PI3K/AKT and JNK pathways. Brazilian Journal of Medical and Biological Research.

[42] Bourke CD, Prendergast CT, Sanin DE (2015). et al. Epidermal keratinocytes initiate wound healing and pro-inflammatory immune responses following percutaneous schistosome infection. Int J Parasitol.

[43] Nedoszytko B, Sokolowska-Wojdylo M, Ruckemann-Dziurdzinska K (2014). et al. Chemokines and cytokines network in the pathogenesis of the inflammatory skin diseases: Atopic dermatitis, psoriasis and skin mastocytosis. Postepy Dermatol Alergol.

[44] Li D, Li XI, Wang A (2015). et al. Microrna-31 promotes skin wound healing by enhancing keratinocyte proliferation and migration. J Invest Dermatol.

[45] Herrling T, Jung K, Fuchs J (2007). UV-generated free radicals (FR) in skin and hair - their formation, action, elimination and prevention ageneral view. SÖFW-Journal.

[46] Oh SJ, Oh Y, Ryu IW (2016). et al. Protective properties of ginsenoside Rb3 against UV-B radiation-induced oxidative stress in HaCaT keratinocytes. Biosci Biotechnol Biochem.

[47] Pastore S, Potapovich A, Kostyuk V (2009). et al. Plant polyphenols effectively protect HaCaT cells from ultraviolet C-triggered necrosis and suppress inflammatory chemokine expression. Ann N Y Acad Sci.

[48] Ighodaro OM, Ebuehi OAT (2009). Aqueous leaf extract of Ocimum gratissimum potentiates activities of plasma and hepatic antioxidant enzymes in rats. Nig Q J Hosp Med.

[49] Chiu CC, Huang CY, Chen TY (2012). et al. Beneficial Effects of Ocimum gratissimum Aqueous Extract on Rats with CCl(4)-Induced Acute Liver Injury. Evid Based Complement Alternat Med.

[50] Gurtner GC, Werner S, Barrandon Y (2008). et al. Wound repair and regeneration. Nature.

[51] Coulombe PA (2003). Wound epithelialization: accelerating the pace of discovery. J Invest Dermatol.

[52] Pastar I, Stojadinovic O, Yin NC (2014). et al. Epithelialization in Wound Healing: A Comprehensive Review. Adv Wound Care (New Rochelle).

[53] Takada K, Komine-Aizawa S, Hirohata N (2017). et al. Poly I:C induces collective migration of HaCaT keratinocytes via IL-8. BMC Immunology.

[54] Wang FP, Li L, Li J (2013). et al. High mobility group box-1 promotes the proliferation and migration of hepatic stellate cells via TLR4-dependent signal pathways of PI3K/Akt and JNK. PLoS One.

[55] Evwierhurhoma E, Ugwu MC, Eze CO (2015). et al. Antibacterial Evaluation of Aqueous and Ethanol Extracts of Ocimum gratissimum and Carica papaya. Annual Research & Review in Biology.

